# Acaricide residues in beeswax. Implications in honey, brood and honeybee

**DOI:** 10.1007/s10661-023-11047-6

**Published:** 2023-03-09

**Authors:** Beatriz Albero, Esther Miguel, Ana I. García-Valcárcel

**Affiliations:** Department of Environment and Agronomy, National Centre for Agricultural and Food Research and Technology, Superior Council of Scientific Research INIA-CSIC, Carretera de La Coruña Km 7.5, 28040 Madrid, Spain

**Keywords:** Varroa spp, Bee colonies, Miticides, Contamination, Apiary location

## Abstract

For beekeeping to be sustainable, the management of colonies for the production of bee products must be economically viable without endangering the lives of bees, and must include acceptable practices such as the treatment of hives with appropriate products. Occasionally, the use of acaricides to treat the hives against varroosis is uncontrolled and can accumulate in the hives, putting the colonies at risk. In this work, a screening of seven acaricides was carried out in different apiaries in Andalusia (Spain). Their distribution in beeswax, brood, honey, and bees from colonies in different surroundings was evaluated at different times. It was found that beeswax was highly contaminated but honey, brood and bees had acceptable levels, below their respective MRL or LD50, after a certain period following varrocide treatments. Acaricides banned for their use against Varroa, such as chlorfenvinphos, cypermethrin and especially acrinathrin, were found in the hives analysed.

**Supplementary Information:**

The online version contains supplementary material available at 10.1007/s10661-023-11047-6.

## Introduction

Beeswax is a natural product consisting of a mixture of lipophilic compounds, which are constructed and formed into a hexagonal shape by the mandibles of worker honeybees (Bogdanov, 2004) to build the combs for honey and pollen storage and brood cradle. The production of beeswax is generally not the aim of beekeepers, so wax sheet foundations made from recycled old combs are provided for honeybees to extend and form the cells. However, beeswax can remain in the hive for several years without being replaced and, due to its lipophilic character, can accumulate non-polar pesticides from various sources such as agriculture and veterinary treatment (Chauzat & Faucon, 2007; Mullin et al., 2010).

Varroa destructor is a year-round ectoparasite that feeds on the haemolymph of larval and adult honeybees. This parasite has spread worldwide and is considered a major threat to apiculture. It is believed to be one of the main causes of colony losses. Therefore, it is necessary to treat the hives against varroosis, because without treatment the colony will die within 3 to 5 years (Gregorc et al., 2018). Organic acids such as oxalic or formic acid or plant essential oils, such as thymol, are used to control Varroa. However, synthetic acaricides such as amitraz, fluvalinate and coumaphos are the most frequently applied to control the mite. But continuous use of these chemicals in the same hive can cause the collapse of honeybee colonies (Rial-Otero et al., 2007). The chemical structure of the acaricide determines its physicochemical properties, such as solubility, molecular mass and lipophilicity, which affect its accumulation in beeswax or in other parts of the hive. Water-soluble acaricides tend to accumulate in honey, whereas lipophilic acaricides tend to accumulate in beeswax. The application of acaricides in the hives can be by direct application of a drip solution, by fumigation or by strips impregnated with the acaricide and placed in the comb frames of a hive. These apicultural practices may result in the accumulation of acaricides in beeswax and in other beekeeping products by diffusion or by migration from beeswax in contact with them. In addition, Varroa resistance to acaricides could occur (Benito-Murcia et al., 2021; Rinkevich, 2020). Recycling of beeswax may not be sufficient to remove all the chemicals accumulated in it, as they resist the melting temperature (Ravoet et al., 2015). Acaricides will remain in the beeswax foundation sheets and will re-enter the hive so a new acaricide application could be toxic to the proper development of the colony.

While maximum residue limits (MRLs) in honey are set by European regulation (EU Commission, 2023) for certain acaricides; 0.05 mg/kg for acrinathrin and cypermethrin; 0.2 mg/kg for amitraz, 0.1 mg/kg for coumaphos and 0.01 mg/kg for chlorfenvinphos, there are no MRL for beeswax despite its use in food and pharmaceutical products. In Europe, there is no quality control of beeswax related to its use.

There are many studies on acaricide levels on beeswax from different parts of the world (Calatayud-Vernich et al., 2017; El Agrebi et al., 2020; Kast et al., 2021; Lozano et al., 2019; Mullin et al., 2010; Perugini et al., 2018) but fewer studies evaluate their distribution or possible transfer from wax to the rest of the hive (honey, brood and bee) (Kast & Kilchenmann, 2022; Murcia Morales et al., 2020) and their relation with the hive environment (Fulton et al., 2019; Calatayud-Vernich et al., 2018). Therefore, the aims of this work were to obtain an overview of the acaricides found in hives in apiaries from Andalusia (Spain), to determine their concentrations in beeswax and other hive components (brood, honey, and bee) at different times, and also to evaluate the possible influence of the environment (agricultural, urban and forest) surrounding the colonies on the acaricide levels. The acaricides analysed were, coumaphos and its metabolite chlorferon, chlorfenvinphos, acrinathrin, cypermethrin and amitraz. Coumaphos and chlorfenvinphos are organophosphate compounds, acrinathrin and cypermethrin are pyrethroids and amitraz is an amidine. As amitraz is usually undetectable in beeswax and honey samples due to its short half-life in these matrices (Shimshoni et al., 2019), this compound was analysed via its metabolites N-(2,4-dimethylphenyl)-formamide (DMF) and N-(2,4-dimethylphenyl)-N-methylformamidine) (DMPF).

## Materials and methods

### Origin of the samples

**Fig. 1:**
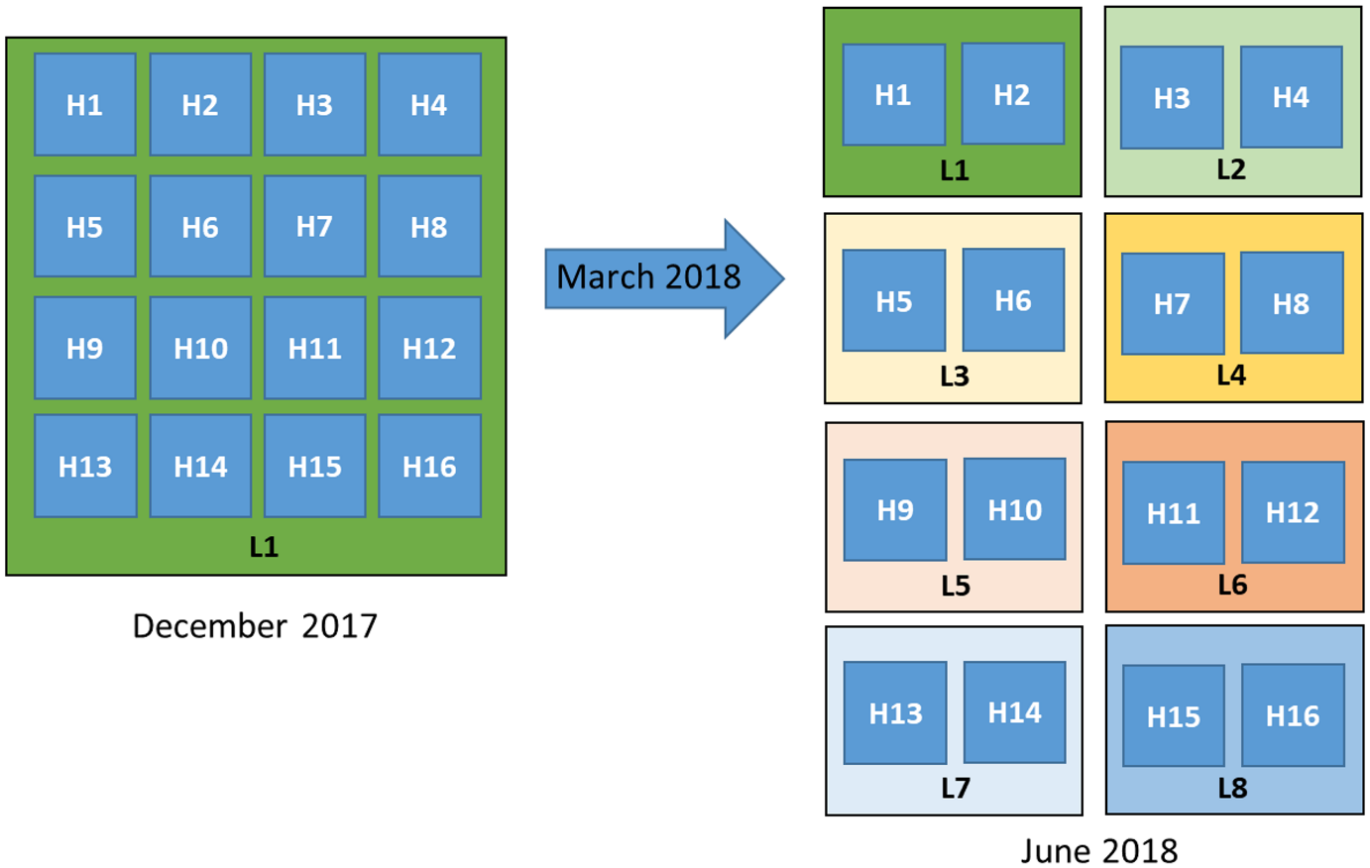
Scheme of the sampling of hives in December and June according to their location

Sampling was performed in sixteen honeybee hives (H1-H16) initially located in the same apiary in Andalusia, Spain (L1) (December 2017). In March 2018, these hives were moved to seven different locations (2 hives in each location), leaving two hives in the original location L1 (Fig. 1). Land use at locations L1-L8 is described in Table 1. In June 2018, a second sampling was conducted in the hives sited at their new locations. Samples of wax, honey, brood, and honeybees were collected from each colony by the beekeepers in December 2017 and June 2018. Approximately, 20 broods, 100 honeybees and 5 g of honey, were collected from each hive. The brood samples for this study were at the larval stage, with a development time of six days. All the samples were stored at -20 °C until analysis.

**Table 1:** Land use of the locations where apiaries were placed in March 2018

Location	Hives	Apiary environment^a^	Land use
L1	H1-H2	Mountainous landscape. Mediterranean-continental vegetation, pines, holm oaks	Forest
L2	H3-H4	Urban landscape: ornamental garden	Urban
L3	H5-H6	Dryland crops: olive and Mediterranean vegetation, near a highroad	Farming
L4	H7-H8	Grassland, scrub with a stream and a highroad in the proximity	Forest-pastureland
L5	H9-H10	Irrigations crops: citrus	Farming
L6	H11-H12	Riparian vegetation and dryland crops: olive	Farming
L7	H13-H14	Dryland crops: olive. Mountainous landscape: pines and holm oaks	Agroforestry
L8	H15-H16	Rural grassland within the city and near a large river	Urban–rural

^a^Source: SIGA (Geographic Information System for Agricultural Data), https://sig.mapama.gob.es/siga/

### Reagents and materials

All pesticide standards were obtained from Sigma-Aldrich (St. Louis, USA): coumaphos, acrinathrin, chlorfenvinphos, cypermethrin and DMPF with purity ≥ 98%; and chlorferon and DMF with 97% purity. Individual stock solutions (1000 mg/L) were prepared in acetonitrile and stored in amber vials at − 20 °C. Individual standard solutions and mixed standard solutions, used for the optimisation and calibration respectively, were prepared by an appropriate dilution of the stock standard solutions with acetonitrile.

Magnesium sulphate (MgSO_4_), sodium acetate (NaAc), formic acid and ammonium formate were obtained from Sigma-Aldrich (Steinheim, Germany). PSA and C18 sorbents were purchased from Scharlab S.L (Barcelona, Spain), and Z-Sep from Agilent (Santa Clara, CA, USA). LiChrosolv acetonitrile was provided by Merck (Darmstadt, Germany). High purity water was obtained using a Milli-Q water purification system (Millipore, Milford, MA, USA).

### Extraction procedure

#### Beeswax

Beeswax samples were free of beebread, honey, cocoons, and brood before their analysis. The beeswax extraction method was based on Niell et al. (2014). Briefly, beeswax pieces of 15 cm^2^ were cut as small as possible and mixed to make a homogenised sample. Then, 1 g of beeswax and 5 mL of acetonitrile were added to a PP (polypropylene) centrifuge tube and the tube was heated in a water bath at 80 °C. When the beeswax had melted, the contents of the tube were homogenised by vortexing for 30 s, followed by sonication for 5 min in an ultrasound water bath at 60 °C. These procedures were repeated five times to ensure an efficient extraction of the pesticides. The sample was then centrifuged at -4 °C and 5000 rpm for 15 min. The supernatant was collected in a PP tube and stored in a freezer at -20 °C overnight, followed by centrifugation to ensure a good separation of the beeswax and the solvent. The supernatant was diluted with acetonitrile (1:1, v/v). An aliquot of the extract was then purified with PSA and C18 (50 mg of each sorbent per ml of extract). The tube containing the sorbents was vortexed for 3 min and centrifuged at 5000 rpm for 10 min. Finally, the supernatant was filtered through a 0.22 µm nylon filter and 1 mL of the filtrate was mixed with 0.01 mL of a solution of acetonitrile with 5% of formic acid in a vial before chromatographic analysis.

#### Honeybee and brood

Sample preparation of honeybees and brood was performed using a modified ultrasound-assisted QuEChERS method (García-Valcárcel et al., 2019). One gram of sample (honeybee or brood) and 5 mL of MilliQ water were added to a centrifuge tube and allowed to stand for 5 min. Honeybees or brood in the tube were then fragmented by shaking in a horizontal shaker with two agate balls and 5 mL of acetonitrile for 10 min. Fragmentation was followed by sonication for 5 min, and a mixture of 4 g of MgSO_4_:NaAc (4:1, w/w) was added to the tube. The tube was vortexed vigorously for 1 min and centrifuged at 5000 rpm for 5 min at 4 ˚C. An aliquot of the supernatant was transferred to a tube containing C18 and PSA in the case of brood (50 mg of each sorbent per ml of supernatant) and MgSO_4_ (150 mg/ml) and PSA (100 mg/ml) in the case of honeybees. The tube was vortexed for 1 min and centrifuged at 5000 rpm at -4 ˚C for 5 min. The supernatant was filtered through a 0.22 μm nylon filter. In a vial, 1 mL of the filtrate and 0.01 mL of a solution of acetonitrile with 5% of formic acid were mixed before quantification by chromatographic analysis.

#### Honey

Honey (1 g) and 7 mL of MilliQ water were vortexed for 1 min in a centrifuge tube. Then, 10 mL of acetonitrile containing 5% of formic acid were added to the tube and shaken by hand, followed by sonication in an ultrasonic water bath for 10 min. A mixture of 4 g of MgSO_4_:NaAc (4:1, w/w) was added and shaken for 30 s followed by centrifugation at 5000 rpm and 4 °C for 15 min. An aliquot of the extract was then cleaned up by adding 0.15 g MgSO_4_, 0.05 g of PSA and 0.05 g of Z-Sep per ml of extract. The extract was then filtered through a 0.22 μm nylon filter before chromatographic analysis.

### Quantification analysis

Quantification of acrinathrin and cypermethrin in the different samples was performed by gas chromatography-mass spectrometry (GC–MS) and by liquid chromatography tandem mass spectrometry (LC–MS/MS) for the remaining acaricides. The same extract was analysed by gas and liquid chromatography.

Analysis by GC–MS was performed in a gas chromatograph (Agilent 7890A) coupled to a mass spectrometer (HP 5977A) equipped with an automatic injector. Separations were performed by using a HP-5MS column, (30 m × 0.25 mm i.d. 0.25 μm film thickness) from Agilent (Torrance, CA, USA). Helium (purity 99.995%) was used as carrier gas at a flow rate of 1.4 mL/min. Operating conditions were as follows: 2 μL extracts were injected into a single-tapered glass liner with deactivated glass wool, with the autosampler in pulsed splitless mode (40 psi for 0.75 min). The injection port temperature was 250 °C. The column temperature was held at 70 °C for 1 min, then programmed at 30 °C/min to 280 °C (held for 7 min) and then at 40 °C/min to 300 °C. The total analysis time was 15.5 min. The mass spectrometric detector was operated in electron impact ionisation mode with an ionisation energy of 70 eV. Ion source and transfer line temperatures were 230 and 280 °C, respectively. Retention time and mass spectra of all analytes were acquired in the full scan mode (mass range from 50 to 600 m/z). Selected Ion Monitoring (SIM) mode was employed for quantitative analysis, using one target and two qualifier ions to identify each analyte. Table S1 lists the mass spectrometric parameters and retention times of acrinathrin and cypermethrin by GC–MS.

An Agilent liquid chromatograph (HPLC 1200 series) equipped with an automatic injector, a degasser, a quaternary pump and a column oven combined with an Agilent 6410 triple quadrupole (QQQ) mass spectrometer with an electrospray ionisation interface (Agilent Technologies, Waldbronn, Germany) was used for the analysis of chlorferon, coumaphos, chlorfenvinphos, DMPF and DMF. The electrospray ionisation source was operated in positive mode with a gas temperature of 300 °C, a gas flow of 9 L/min, a nebuliser pressure of 35 psi and a capillary voltage of 4000 V. Nitrogen was used in the nebuliser and in the collision cell. The chromatographic column was EVO-C18 of 100 × 3 mm i.d. and 2.6 μm, 100 Å particle size (Phenomenex, Torrance, CA, USA). The mobile phase consisted of A (acetonitrile with 0.1% formic acid) and B (ammonium formate 5 mM), the flow rate was of 0.4 mL/min and the injection volume was 10 μL. The gradient elution programme used was as follows: starting at 50% of solvent A, increased to 80% in 10 min, to 95% in 3 min and kept constant for 2 min. Return to initial conditions in 7 min and keep constant for 8 min. The QQQ mass spectrometer was operated in selected reaction monitoring (SRM) mode and two optimised SRM transitions were monitored for each acaricide (Table S2). Data were processed using the Mass Hunter Workstation software for qualitative and quantitative analysis (Agilent Technologies, Palo Alto, CA, USA).

Matrix-matched external calibration was used to quantify the acaricides in honeybees, brood, and honey. However, as it was difficult to find a wax sample without the selected pesticides, a standard addition method was employed to quantify pesticides in beeswax by adding different concentrations (0, 20, 100 and 500 ng/mL) of the target analytes to different aliquots of the same beeswax extract. In addition, the standard addition method could compensate for matrix effects due to the wax.

The method LOQ (limit of quantification) was set as the minimum concentration that can be quantified with acceptable accuracy (between 70–120%) and good precision (RSD < 20%). Due to the difficulty in finding blank beeswax samples, these samples were analysed, and the target pesticides found in them were considered to calculate the LOQ, which should be the lowest spiked level validated to meet the SANTE criteria (SANTE, 2021).

Recovery and precision of the methods were carried out at two levels, 2 and 20 µg/kg for brood, honey, and bees and at 10 and 1000 µg/kg for wax. Four replicates of each concentration level were performed for recovery testing. Repeatability and reproducibility were evaluated for both spiking levels; over 1 and 4 days, respectively. Linearity was assessed by spiking blank extracts with a standard solution at six concentration levels (from 2 to 1000 µg/kg). If the area of a positive finding was above the linear range, the sample was diluted to an appropriate concentration and reinjected in a new curve.

### Acaricide risk assessment

Hazard quotients (HQ) in wax were determined as the sum of all acaricide residues detected in wax (µg/kg) divided by their respective contact LD_50_ (µg/bee) in each beeswax sample (OECD, 2018). LD_50_ values were taken from Sánchez-Bayo and Goka (2014) and PPDB/VSDB (Pesticide Properties DataBase /Veterinary Substances DataBase). The risk to honeybees and brood was evaluated by comparing the LD_50_ with the residue levels for each acaricide found in bees and brood. Contamination of honey was assessed by comparing the acaricide levels in honey with their MRLs.

## Results

### Method validation

The LOQ was 2 µg/kg for all compounds in honey, brood, and honeybee. Recoveries for brood, honey and honeybee ranged from 71 to 108% with good precision, RSD between 1 and 19% (repeatability) and between 2 and 21% for reproducibility. Recoveries in wax ranged from 80–110% with RSD between 3 and 17% (repeatability) and 5–20% (reproducibility). The LOQ in beeswax was 10 µg/kg for all compounds. Matrix-matched calibration curves showed good correlation coefficients R^2^ > 0.98 in the range of 2–1000 µg/kg.

### Acaricide residues in beeswax

#### Levels in beeswax in December and June

Of the total acaricides analysed in all wax collected in December 2017, coumaphos was the most abundant followed by DMF, acrinathrin, chlorfenvinphos and DMPF with percentages of 52%, 28%, 11%, 4% and 3%, respectively. The remaining compounds analysed in wax were present in percentages lower than 3%.

Considering the total compounds analysed in all waxes at the different locations in June 2018, the most abundant acaricide was coumaphos with 33% of the total acaricides, followed by acrinathrin with 32%, DMF with 25% and chlorfenvinphos with 7%. Chlorferon, DMPF and cypermethrin represented less than 5% of the total.

Coumaphos, amitraz (as DMF and DMPF), chlorfenvinphos and acrinathrin were present in all the waxes collected in December, chlorferon was found in 87% and cypermethrin in 31% of the beeswax samples collected in December (Table 2). For the positive compounds found in waxes in December, the ranges of concentrations found were very wide, indicating a high inter-hive variation, although all waxes were collected from hives located at the same site and sampled at the same time. The mean concentration of coumaphos was about twice that of amitraz and about six times that of acrinathrin for the set of waxes where these compounds were quantified (Table2).

**Table 2:** Summary of compounds quantified in beeswax (n = 16) at the initial location in December 2017 and at the final locations in June 2018^a^

Compounds	Positive (%)		Minimum (µg/kg)		Maximum (µg/kg)		Mean (µg/kg)		Median (µg/kg)	
	Dec.	June	Dec.	June	Dec.	June	Dec.	June	Dec.	June
Chlorferon	87	81	24	22	1532	174	225	76	112	66
Coumaphos	100	100	323	255	35,678	6687	5958	1617	3506	807
Chlorfenvinphos	100	100	55	46	1409	1845	445	315	401	176
Amitraz (DMF)	100	75	374	28	24,880	17,799	3210	1597	714	134
Amitraz (DMPF)	100	69	29	10	3266	1298	322	159	95	48
Acrinathrin	100	100	120	107	4278	4447	1214	1541	687	553
Cypermethrin	31	0	62		93		78		81	

^a^Minimun, maximun, mean and median values of positive samples (> LOQ)

Coumaphos, chlorfenvinphos and acrinathrin were present in all the waxes collected in June. DMF and DMPF (amitraz metabolites) were present in 75% and 69% of the samples, respectively. Chlorferon was present in 81% of the samples and cypermethrin was not quantified in any wax sample at any location in June 2018. The mean concentrations of coumaphos, DMF and acrinathrin were similar, between 1.5 and 1.6 mg/kg, when considering positive samples for these acaricides (Table 2).

The level of chlorferon was always lower than that of coumaphos in the same wax sample and the level of DMF was higher than that of DMPF within the same wax. However, no correlation was found between chlorferon and coumaphos or DMF and DMPF.

Considering the average of the 16 wax samples in December and in June, a decrease in the concentration of each acaricide was observed over time (Table 2). The most pronounced decrease was observed for coumaphos, followed by amitraz with a decrease of 75% and 63%, respectively, with respect to their initial mean values. On the other hand, mean concentrations of chlorfenvinphos were similar at both sampling times and mean concentrations of acrinathrin were slightly higher in June. Nevertheless, considering the median values, all acaricides values decreased with time. The mean of total acaricide load in the total waxes sampled in December was 11.4 mg/kg while in those sampled in June it was 4.8 mg/kg, in other words, six months later the total pesticide load represented a 42% of the load found in December.

#### Levels in the different locations

**Fig. 2:**
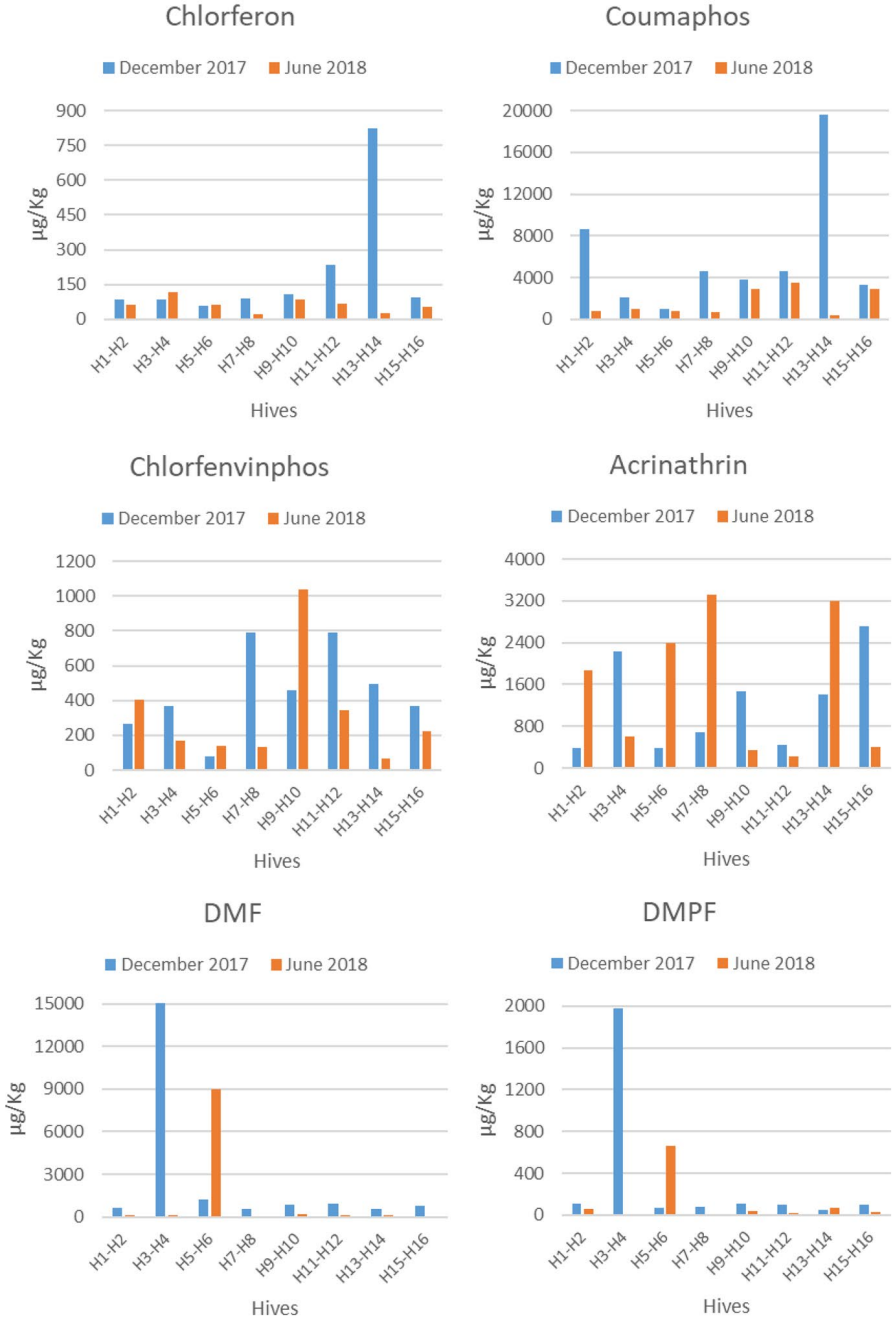
Levels of acaricides (µg/kg) in beeswax in December in hives (H1-H16) at location L1 (blue colour) and in June in the same hives (H1-H16) at the different locations (from L1 to L8) (orange colour)

The differences in acaricide concentrations for the same hives at the initial location in December and at the final locations in June are shown in Fig. 2. Cypermethrin was not present in any of the beeswax samples collected in June and was only detected in five hives in December with a low mean concentration level of 24 µg/kg, so this compound is not shown in the Fig. 2.

The acaricide with the highest concentration in most of the waxes analysed in both December and June, was coumaphos (coumaphos + chlorferon). The highest losses of coumaphos from the initial levels in December occurred at sites L1 (hives H1-H2), L4 (hives H7-H8), and L7 (H13-H14), where only 2–14% of the initial concentration of coumaphos remained in beeswax. Chlorfenvinphos levels in June increased between 1.5 and 2.2 times in sites L1 (H1-H2), L3 (H5-H6) and L5 (H9-H10), while levels decreased in the remaining hives. Acrinathrin residues increased sharply in June in 50% of the trial sites (sites L1 (H1-H2), L3 (H5-H6), L4 (H7-H8) and L7 (H13-H14)). Amitraz (DMF + DMPF) levels decreased between 99 and 67% of the initial level at all sites except at site L3 (H5-H6) where amitraz increased sevenfold the initial level in December.

**Fig. 3:**
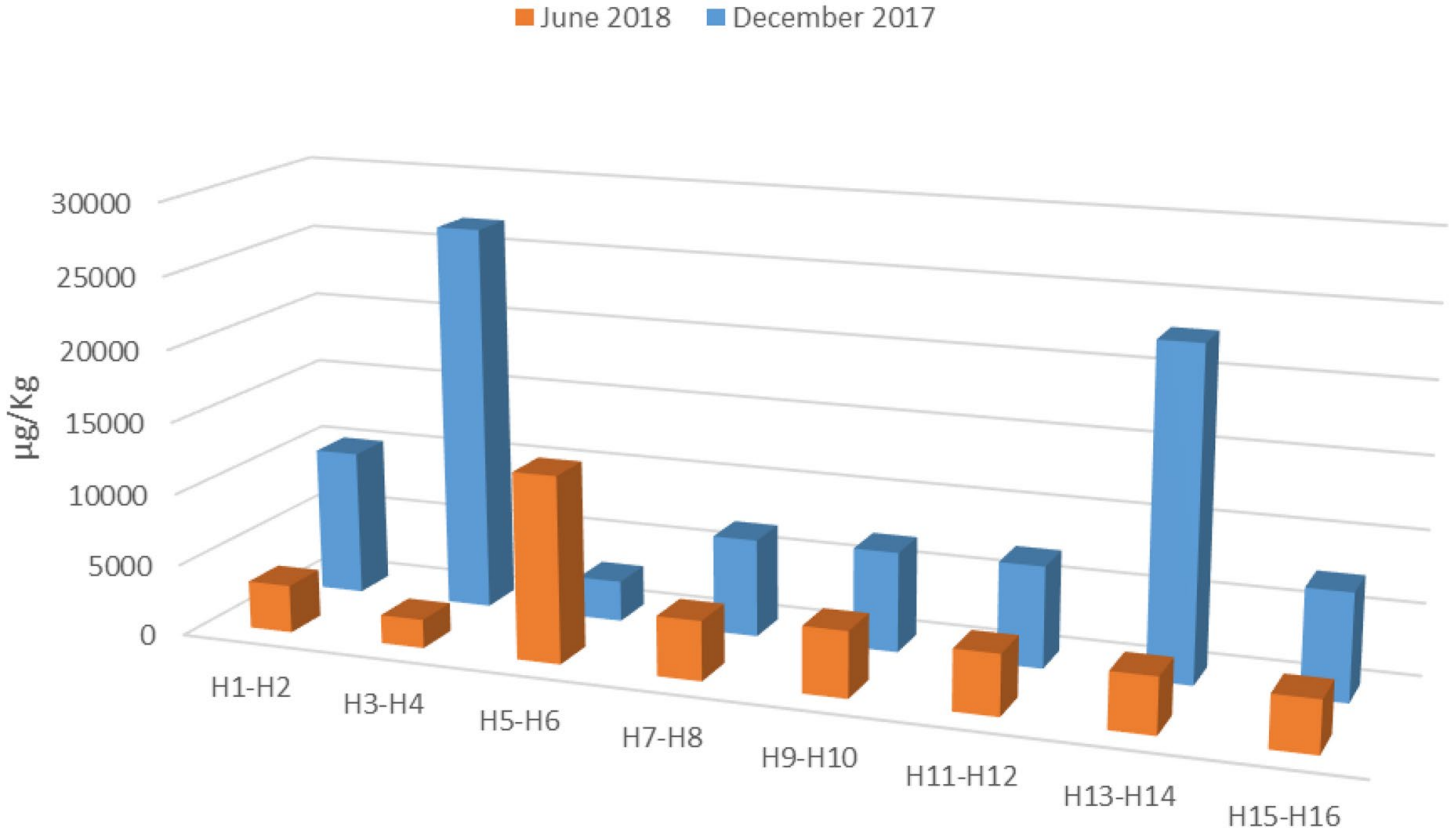
Total acaricide load in beeswax in December and June

With the exception of beeswax at site L3 (H5-H6), the total acaricide load decreased over time at each location (Fig. 3). At this site (L3), a significant increase in the amount of amitraz and acrinathrin and, to a lesser extent, of chlorfenvinphos was observed in relation to their content in December (Fig. 2).

#### Pesticide risk assessment in waxes

**Fig. 4:**
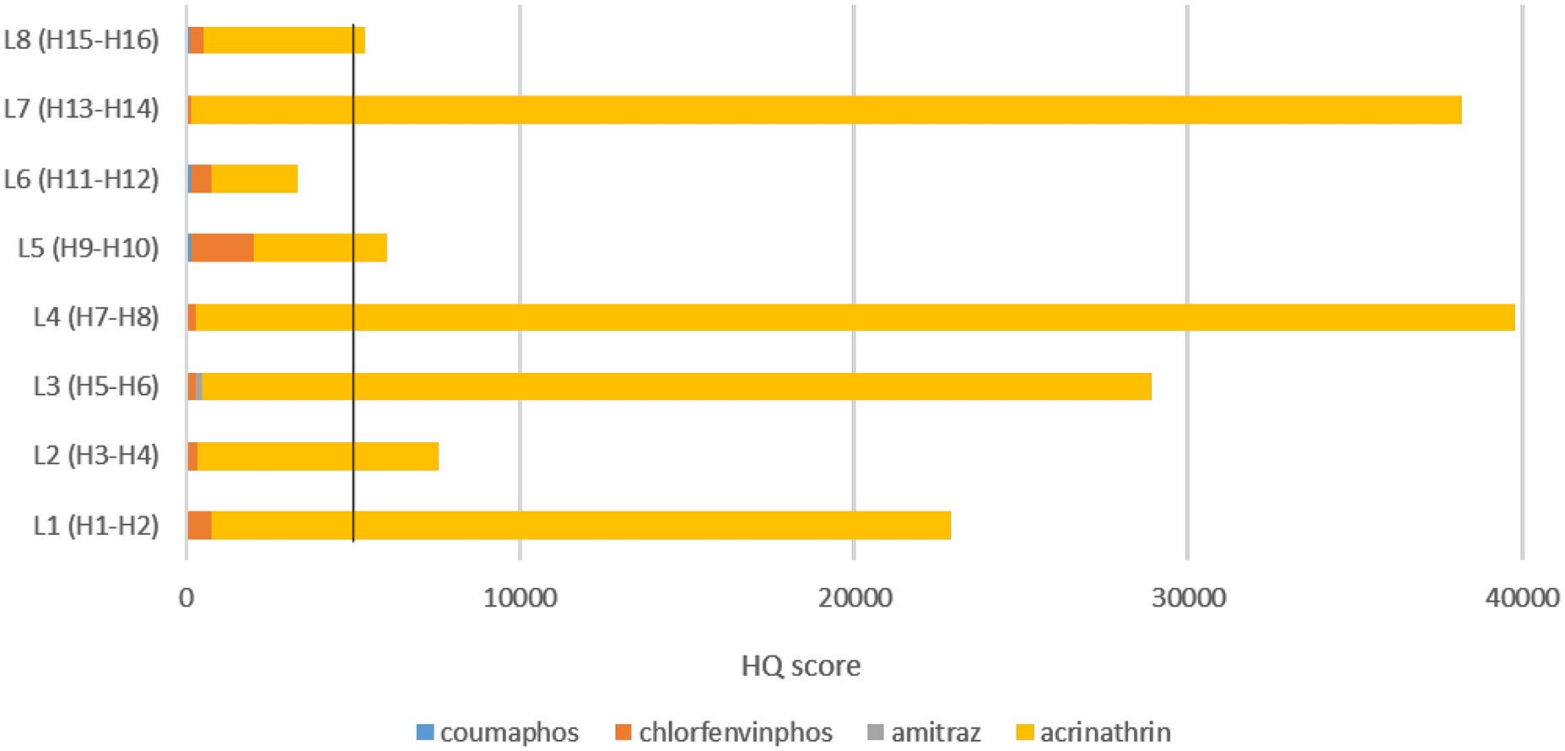
Significance of the different acaricides on the HQ value according to the location of the hives in June

Due to the high toxicity of acrinathrin to adult honeybees, contact LD_50_ = 0.084 µg/bee, this compound contributes the most to the HQ score (Fig. 4), whereas coumaphos with a LD_50_ = 20 µg/bee and amitraz with a LD_50_ = 50 µg/bee are the acaricides that contribute less to the HQ score. Chlorfenvinphos, despite its low wax levels, also contributes to an increase in HQ due to its LD_50_ = 0.55 µg/bee. HQ values in wax were often > 5000 (Fig. 4). Beeswax from locations L4 and L7 showed the highest concentrations of acrinathrin.

### Acaricide residues in honey, brood and bees

#### Levels of acaricides in December and June

Of the seven acaricides analysed, none of the honey, brood or bee samples contained cypermethrin or chlorfenvinphos. Acrinathrin was only quantified in one honey sample (24.5 µg/kg in H15) and one brood sample (14.4 µg/kg in H3) at the original location in December. These hives contained the highest concentration of acrinathrin in wax (Fig. 2). Chlorferon was quantified in 3 of the 16 honey samples (H2, H8 and H10) and in one bee sample (H15) at the original site, but not in the brood. This compound was also quantified in one of the 16 honey samples at the exposure locations (H11). In December, coumaphos was present in 69%, 25% and 25% of honey, brood and bee samples, respectively, whereas in June its presence decreased to 19% and 6%, in honey and bees, respectively, and increased up to 50% in brood. Amitraz (DMF + DMPF) was present in all honey and bee samples analysed and in 87% of the brood at the initial site L1 and in June, its presence decreased to 94%, 7% and 12% in honey, brood and bees, respectively.

Table 3 summarises the mean concentrations of coumaphos and amitraz in the hives analysed at the initial and final sites. Honey was the matrix with the highest amount of both acaricides in December (initial site) and June (exposure sites). Concentrations of amitraz were higher than those of coumaphos for both sampling times and for all matrices. Levels of coumaphos and amitraz were higher in the initial site than in the exposure locations for all three matrices, except for brood where the mean amount of coumaphos was similar in the initial and final apiaries (Table 3). At the initial site, amitraz levels were higher in bees than in brood, but at the exposure sites, similar levels of amitraz were found in bees and brood with very low levels.

**Table 3:** Mean concentrations (µg/kg) of coumaphos and amitraz in honey, brood, bees and wax in hives placed at the different sites in December and June

	Coumaphos								Amitraz							
	Honey		Brood		Bee		Wax		Honey		Brood		Bee		Wax	
Hives^a^	Dec.	June	Dec.	June	Dec.	June	Dec.	June	Dec.	June	Dec.	June	Dec.	June	Dec.	June
H1-H2	11.5	n.d^b^	n.d	5.6	n.d	n.d	8665	800	32	73	15	n.d	63	n.d	728	196
H3-H4	14.2	n.d	2.4	2.5	3.2	n.d	2054	1017	2195	24	36.4	n.d	93	n.d	22179	87
H5-H6	4.4	n.d	9.7	2.0	1.8	n.d	1011	761	571	9	32.5	2.9	387	2.7	1305	9619
H7-H8	3.4	n.d	2.3	1.5	n.d	n.d	4595	661	171	11	9.1	n.d	50	n.d	649	n.d
H9-H10	12.6	5.6	n.d	n.d	n.d	2.25	3857	2918	72	23	6.2	n.d	138	3.2	990	210
H11-H12	8.9	15.7	n.d	1.2	1.0	n.d	4577	3472	32	25	8.4	n.d	94	n.d	993	113
H13-H14	0.0	n.d	n.d	5.9	n.d	n.d	19548	416	32	122	5.3	n.d	100	n.d	571	189
H15-H16	9.7	n.d	n.d	1.0	3.0	n.d	3352	2892	64	24	35.4	n.d	78	n.d	847	41
Mean^c^	8.1	2.7	1.8	2.5	1.1	0.3	5957	1617	396	39	18.5	0.4	125	0.7	3533	1307

^a^For information on the location of the hives at December and June see Fig. 1 or Table 1

^b^n.d.: not detected (< LOD)

^c^If the acaricide, coumaphos or amitraz (DMF + DMPF), was not detected in a sample it was considered 0 for the determination of mean

#### Influence of the exposure location

The highest concentration of coumaphos at the initial site was 14.2 µg/kg for honey, followed by brood (9.7 µg/kg) and bees (3.2 µg/kg). At the June exposure sites, the concentration of coumaphos was generally lower than at the initial site, with some exceptions: at location L6 (H11-H12) an increase was observed in honey and brood with respect to the initial values (Table 3), and at location L7 (H13-H14), a significant increase was noted in brood (from not detected to 5.9 µg/kg). In addition, in hives H9-H10, coumaphos was quantified in bees in June but not in December. No correlation was found between coumaphos levels in wax and the other matrices.

In December, when all hives were at location L1, all matrices contained amitraz, with the highest concentrations in honey and the lowest in brood. While in June, amitraz was found in all honey samples, at lower levels than in December, but found only in brood samples from one site and in bee samples from two sites. It is necessary to point out the high levels in honey from hives (H3-H4) and (H5-H6) collected in December, with levels exceeding the MRL of 200 µg/kg for amitraz in honey. In addition, bees contained relatively high concentrations of amitraz in the initial apiary (Table 3). The highest amitraz concentrations in beeswax in December (> 1.2 mg/kg) correspond to the highest initial levels of this compound in honey, brood and bees.

**Fig. 5:**
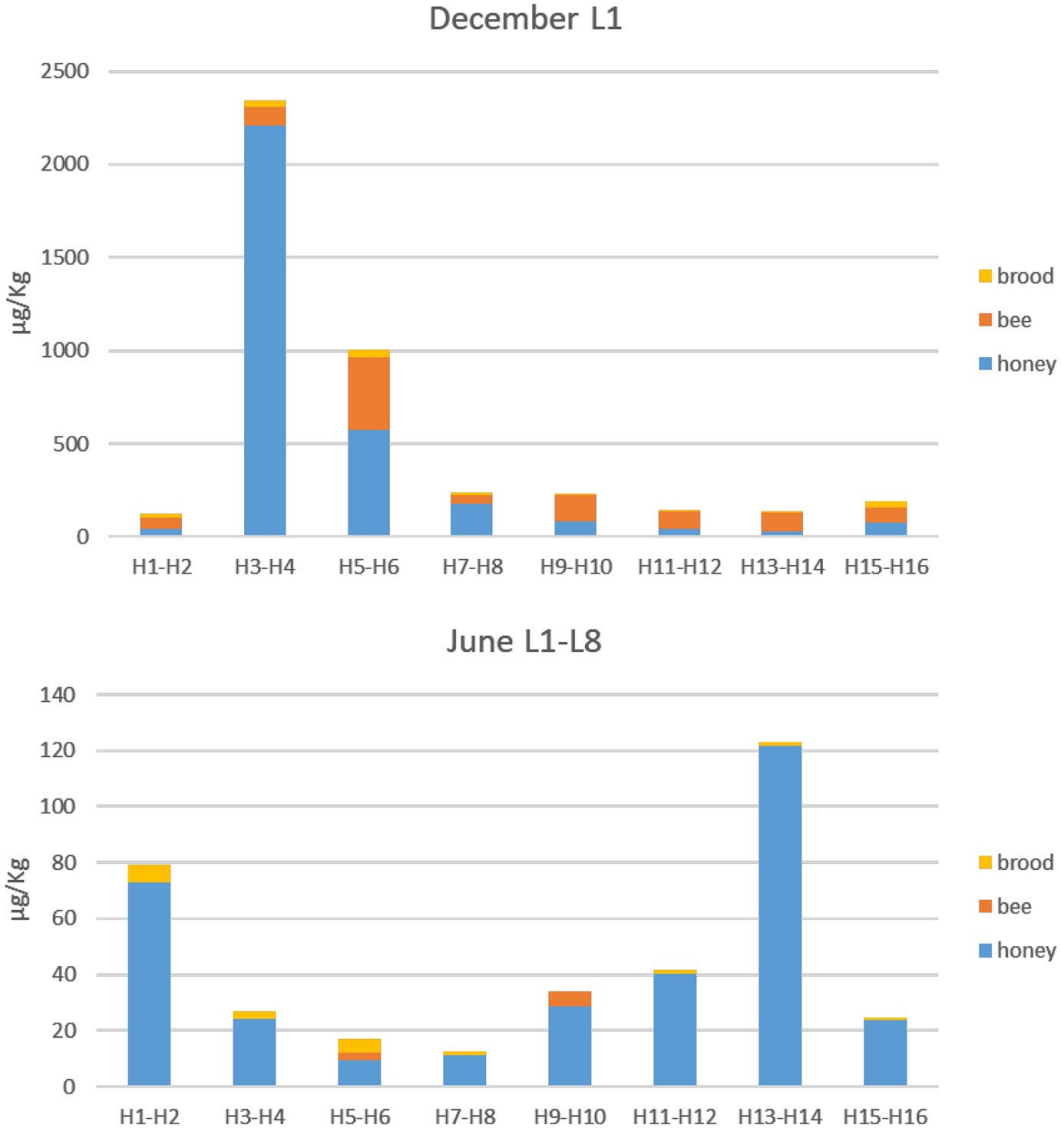
Total load of acaricides in brood, bees and honey sampled from the different hives (H) in December at location L1 and in June at locations L1-L8

In December, the total load of acaricides in honey, bees and brood in most hives was between 120 and 200 µg/kg in most hives, with the exception of the hives H3-H4 and H5-H6 with about 2300 µg/kg and 1000 µg/kg, respectively (Fig. 5). These values were mainly due to the high level of amitraz found in honey at this initial time. In June, the total load was between 12 and 120 µg/kg, which represents a reduction of up to 99% of the initial load in some hives (H3-H4).

#### Potential impact of acaricides in the bee colonies

The highest concentration of coumaphos found in brood was 9.7 µg/kg that corresponds to 1.2 µg/larva (a brood weighs 125 mg) lower than its contact LD50 of 2.7 µg/larva (Dai et al., 2017). For honeybees, 0.25 µg/bee was the highest concentration found (one bee weighs approximately 100 mg), which is lower than its LD50 = 24 µg/bee according to Stoner and Eitzer (2013) or LD50 = 20.29 µg/bee reported by Sánchez-Bayo and Goka (2014). The highest concentrations of amitraz were 4.55 µg/larva and 38.7 µg/bee, which did not exceed their LD50 values (14.83 µg/larva (Dai et al., 2017) or 50 µg/bee (PPDV)). For acrinathrin, no LD50 values were found for larvae, but the concentration of acrinathrin found in a brood sample was 14.4 µg/kg (1.8 µg/larvae), much higher than the LD50 reported in the literature for this compound in adult bees.

## Discussion

### Levels in beeswax in December and June

In Spain, the Royal Decree 608/2006 (Real Decreto, 2006) makes the application of at least one treatment per year (autumn) against varroosis mandatory. In view of the results of the wax sampling in December, it seems that the hives were treated in autumn with coumaphos and, in some cases, with amitraz, judging by the high concentrations of these compounds found and by the percentage of each acaricide found in relation to the total amount of acaricides detected. These treatments (coumaphos and amitraz) are in accordance with the main veterinary treatments in Spanish apiaries (Calatayud-Vernich et al., 2017). The mean concentration of coumaphos (coumaphos + chlorferon) in the 16 hives located in site L1 was 6.0 mg/kg, which is similar to that reported for coumaphos in other works analysing wax from Spanish hives (Calatayud-Vernich et al., 2017, 2018; Murcia Morales et al., 2020). However, the levels of coumaphos in the different hives of this apiary were variable, probably due to the greater or lesser recycling or ageing of the wax. A mean concentration of 0.8 mg/kg was observed for amitraz (DMF + DMPF), which is similar to the concentration level (0.74 mg/kg) reported by El Agrebi et al. (2020). Concentrations in hives H3 and H4 were excluded from this mean because they were too high (Fig. 2), probably due to the application of amitraz in these hives close to the sampling. Chlorfenvinphos and acrinathrin were detected in all the beeswax samples collected in December. These acaricides are not allowed in apiculture as treatments against Varroa, but chlorfenvinphos is used as an acaricide in other livestock species and acrinathrin has been allowed to be used in agriculture until the end of 2021 (EU Commission, 2023). Mean concentrations in wax from this apiary were relatively low, 1.21 mg/kg and 0.44 mg/kg for acrinathrin and chlorfenvinphos, respectively. Therefore, these compounds were most likely already present in the foundation sheets employed in these hives, which were obtained from recycled wax, where they can accumulate and persist long time after their application (Lozano et al., 2019; Mullin et al., 2010). Acaricides can withstand the melting temperature of recycled wax and accumulate in beeswax. The lipophilic character of the compound, together with its half-life in beeswax, determines its potential accumulation in it. The higher lipophilicity of acrinathrin (log Kow = 6.3) compared to other compounds may explain its persistence in wax.

In June, a decrease in the concentration of all acaricides in wax was observed except for acrinathrin (Fig. 2), which increased its residual level in some hives. Although this acaricide is not allowed in apiculture, it is likely that it was applied illegally in these hives between the two samplings. This probable illegal treatment has also been reported in some Spanish apiaries (Jiménez et al., 2005; Orantes-Bermejo et al., 2010; Serra-Bonvehí and Orantes-Bermejo, 2010; Calatayud-Vernich et al., 2018). In December, the detection frequency of cypermethrin was low (20%) with a mean concentration of 0.08 mg/kg. This compound, with a log Kow = 6.6, has a high affinity for wax, so its presence could be due to the use of recycled wax, which did not successfully eliminate cypermethrin during its treatment. Nevertheless, cypermethrin was not found in wax samples in June, probably due to its low presence in December and to its heterogeneous distribution in the wax (Kast et al., 2020), so no wax pieces containing cypermethrin were sampled in the random June sampling.

From December to June, the content of coumaphos (coumaphos + chlorferon) decreased 73%, chlorfenvinphos 30% and amitraz (DMF + DMPF) 64%. Coumaphos and chlorfenvinphos with similar lipophilicity (log Kow of 3.9 and 3.8, respectively) dissipated at different rates. This is most likely due to a recent treatment with coumaphos, whereas chlorfenvinphos was already present in the wax and strongly retained in it before coumaphos was applied. In addition, amitraz, with log Kow of 2.1 and 2.9 for DMF and DMPF, respectively, should have dissipated over time to a greater extent than coumaphos. However, the dissipation of amitraz was slower than that of coumaphos. This apparently contradictory result may be due to a new application of amitraz close to the June sampling, which increased the mean value in that month (Fig. 2).

### Distribution of acaricides in beeswax depending of the location

High spatial and temporal variations in acaricide concentrations in beeswax from different colonies were observed (Fig. 2). In the initial location L1, except for hives H3-H4 and H13-H14 that had a total acaricide load 3–4 times higher than the other hives, the average total load was about 7000 µg/kg (Fig. 3). In the case of hives H3-H4, an extraordinarily high amount of amitraz was found and in the case of hives H13-H14, a high amount of coumaphos was detected (Fig. 2), which could indicate an additional treatment with these acaricides at the initial location, L1. In June, hives placed at L2 and L7 reduced their acaricide load to 9% and 17% of the initial load, respectively. In the remaining hives, the decrease was close to 35%. The faster rate of depletion of amitraz and coumaphos in these hives could be due to a sampling of wax in December, close to the placement of the treatment strips (Kast et al., 2020) or to the geographical location of the hives in June, whose characteristics (weather) facilitate this dissipation.

Acrinathrin, a highly lipophilic compound, was found at higher levels in beeswax from hives located in L1, L3, L4 and L7 in June (from 2.3 to 4.7 times higher) than when there were sited in L1 in December (Fig. 2). Acrinathrin has been employed as a miticide-insecticide in agriculture, which could justify the amounts of this compound in L3 and L7, sites with olive groves, where acrinathrin could have been applied to control the olive fly and transported by honeybees to the hives. Nevertheless, the high amounts (around 3000 mg/kg) found in L1 and L4, surrounded by pine forests and Mediterranean vegetation, suggest the use of this pyrethroid by beekeepers, as already pointed out in another work on Spanish waxes (Calatayud-Vernich et al., 2018).

### HQ wax values for the beeswax samples analysed

HQ wax was only calculated for samples collected in June, when the acaricide load was lower than in the December sampling. Figure 4 shows that at all locations, except L6, hives contained beeswax with HQ > 5000, which is considered a high pesticide load and is associated with queen replacement and higher colony mortality, thus representing an increased toxicity to bees (Traynor et al., 2016). Acrinathrin was the acaricide that contributed most to the high HQ in wax due to its high toxicity (LD_50_ 0.17 µg/bee). Agrebi et al. (2020) provided recommendations for the reuse of beeswax, suggesting that waxes should not be recycled and reused if the HQ is > 5000.

### Acaricides inside the hives

Acaricides found in beeswax can migrate by diffusion/partitioning to other components of the hive such as brood, beebread, and honey. Bees can be contaminated by contact when walking on hive frames and orally by eating beebread or honey that has been in contact with wax, or by chewing beeswax to build the combs (Tremolada et al., 2004; Lozano et al., 2019; Murcia Morales et al., 2020). The rate of migration depends on the solubility of the compound in each beehive matrix. The high prevalence of coumaphos found in honey in December could be due to the proximity of the treatment with this compound to the sampling date, or to bees carrying coumaphos in their bodies and contaminating the honey. Nevertheless, the residue levels detected were in the range of those found in the literature (< 40 µg/kg) (Maver & Poklukar, 2003; Valdovinos-Flores et al., 2016). These levels decreased until they disappeared in almost all honey samples in June. In any case, these levels are below the MRL set for coumaphos in honey, which makes it safe for consumption.

In brood samples collected in December, only a few contained coumaphos, whereas in June, brood contained residues of coumaphos at almost all locations (Table 3). Brood is rich in lipids and coumaphos, as a fat-soluble compound, may accumulate more in this matrix than in honey. Coumaphos can migrate from wax to brood directly by contact or indirectly by migration into the larval diet. Although the concentration levels in brood were similar in December and June, the prevalence of coumaphos was higher in June than in December. This could be explained by a migration of this lipophilic compound from beeswax into the brood after a certain time after its treatment. The levels of coumaphos in brood were much lower than those reported by Bajuk et al. (2017) (100–250 µg/kg) after coumaphos treatment and of the same order as those found by Murcia Morales et al. (2020) in hives not treated with coumaphos (from 1.5 to 12 µg/kg). The migration to brood could adversely affect colony development, although the levels found in the brood were lower than the LD_50_.

Honeybees are in contact with, and therefore exposed to, acaricides applied to the hives. However, the determination of these compounds in bees could be underestimated due to biotransformation and rapid excretion in honeybees, as well as, the possible disorientation of contaminated bees to return to their hives (Calatayud-Vernich et al., 2018). In this study, low concentrations of coumaphos were found in bees, with maximum values in December (mean 1.1 µg/kg).

Amitraz (DMF + DMPF) was found in all honey, brood, and bee samples analysed in December and was the acaricide with the highest concentration of all compounds studied. The highest concentration was found in honey in December, 396 µg/kg, which decreased to 39 µg/kg in June. The high level of this compound in wax indicated that this treatment was applied near December in the hives H3-H4 and probably in H5-H6 hives and then transferred to honey in these hives, reaching levels above the allowed MRL (> 200 µg/kg). The average concentration in bees was 125 µg/kg in December, which practically disappeared in June. According to Mullin et al. (2010), amitraz metabolites bioaccumulate in bees to a greater extent than coumaphos. In brood, amitraz was also present in all the samples analysed in December at concentrations ranging from 8.4 to 36.4 µg/kg, which are similar (from 1.9 to 35 µg/kg) to those found by Murcia Morales et al. (2020) two months after the amitraz application in hives. Amitraz residues in brood and bees decreased to almost disappearance in June, but remained in honey. This could be due to the more hydrophilic nature of honey that would have a greater affinity for DMF and DMPF, which have low Kow values.

## Conclusions

Coumaphos and amitraz were the main acaricides used to control Varroa in the hives analysed, although it appears that acrinathrin was also employed in some hives. The low levels of chlorfenvinphos and cypermethrin found in beeswax suggest that these acaricides came from recycled or reused waxes in the hives, rather than from a recent treatment. Levels of coumaphos and amitraz in beeswax decreased with time after December and were independent of the landscape surrounding the colonies.

There is evidence that contaminated beeswax from the application of coumaphos and amitraz to hives can contaminate honey and brood in contact with it, although the levels found did not reach the LD_50_ for brood or the MRLs for honey, except for amitraz in December in some hives (H3-H4, H5-H6).

The high HQ values obtained for beeswax suggest a possible adverse effect in the bee colony, particularly due to the acrinathrin content, but only one sample out of sixteen brood samples contained it. The non-observed effect concentration (20 mg/kg) for larval exposure to coumaphos in wax was not exceeded by any of the beeswaxes analysed in this study.

In order to achieve a sustainable beekeeping, the condition of the hives should be regularly monitored for pesticide levels. The results of this work show that it is necessary to reduce the load of varrocides in waxes by their correct application and to use only authorised acaricides for Varroa treatment. In addition, alternative treatments against Varroa or products with a less lipophilic character should be developed. Putting these recommendations into practice, together with other beekeeping practices, will provide beekeeping products that benefit the society while preserving ecosystem biological resources, such as plant biodiversity and a healthy bee population.

## Supplementary Information

Below is the link to the electronic supplementary material.

Supplementary file1 (DOCX 13 KB)

## Data Availability

Data will be made available on reasonable request.
