# Supplementary material for: Acaricide residues in beeswax. Implications in honey, brood and honeybee
Source: Environ Monit Assess. 2023 Mar 9;195(4):454. doi: 10.1007/s10661-023-11047-6 (PMC9998570; doi:10.1007/s10661-023-11047-6)
Supplement: Supplementary file 1 — Supplementary file1 (DOCX 13 KB) [file 10661_2023_11047_MOESM1_ESM.docx]

Table S1 Mass spectrometric parameters and retention time by GC-MS

| Pesticide | Target ion | Qualifier ion 1 | Qualifier ion 2 | t_R_ (min) |
| --- | --- | --- | --- | --- |
| Acrinathrin | 208 | 181 | 289 | 9.69 |
| Cypermethrin | 163 | 165 | 181 | 11.31 |

Table S2. Optimized SMR transitions by LC-MS/MS.

| Pesticide | Precursor | Product ions  Quantifier (qualifier) | Fragmentor (V) | Collision energies (V)  Quantifier (qualifier) |
| --- | --- | --- | --- | --- |
| Chlorferon | 211 | 147 (131) | 110 | 40 (25) |
| Coumaphos | 363 | 307 (227) | 110 | 16 (28) |
| Chlorfenvinphos | 359 | 155 (99) | 50 | 10 (20) |
| DMPF | 163 | 122 (107) | 100 | 23 (32) |
| DMF | 150 | 132 (107) | 125 | 16 (28) |
